# A novel major facilitator superfamily-type tripartite efflux system CprABC mediates resistance to polymyxins in *Chryseobacterium* sp. PL22-22A

**DOI:** 10.3389/fmicb.2024.1346340

**Published:** 2024-02-23

**Authors:** Lu Zhang, Miao Wang, Rui Qi, Yilin Yang, Ya Liu, Nianqing Ren, Zihan Feng, Qihao Liu, Guangxiang Cao, Gongli Zong

**Affiliations:** ^1^Biomedical Sciences College & Shandong Medicinal Biotechnology Centre, Shandong First Medical University & Shandong Academy of Medical Sciences, Ji’nan, China; ^2^NHC Key Laboratory of Biotechnology Drugs (Shandong Academy of Medical Sciences), Ji’nan, China; ^3^Shandong Fengjin Biopharmaceuticals Co., Ltd., Yantai, China

**Keywords:** *Chryseobacterium* sp., polymyxins, resistance, MFS transporter, efflux pump inhibitor, baicalin

## Abstract

**Background:**

Polymyxin B (PMB) and polymyxin E (colistin, CST) are polymyxin antibiotics, which are considered last-line therapeutic options against multidrug-resistant Gram-negative bacteria in serious infections. However, there is increasing risk of resistance to antimicrobial drugs. Effective efflux pump inhibitors (EPIs) should be developed to help combat efflux pump-mediated antibiotic resistance.

**Methods:**

*Chryseobacterium* sp. PL22-22A was isolated from aquaculture sewage under selection with 8 mg/L PMB, and then its genome was sequenced using Oxford Nanopore and BGISEQ-500 platforms. *Cpr* (Chryseobacterium Polymyxins Resistance) genes encoding a major facilitator superfamily-type tripartite efflux system, were found in the genome. These genes, and the gene encoding a truncation mutant of CprB from which sequence called CprBc was deleted, were amplified and expressed/co-expressed in *Escherichia coli* DH5α. Minimum inhibitory concentrations (MICs) of polymyxins toward the various *E. coli* heterologous expression strains were tested in the presence of 2–128 mg/L PMB or CST. The pumping activity of CprABC was assessed via structural modeling using Discovery Studio 2.0 software. Moreover, the influence on MICs of baicalin, a novel MFS EPI, was determined, and the effect was analyzed based on homology modeling.

**Results:**

Multidrug-resistant bacterial strain *Chryseobacterium* sp. PL22-22A was isolated in this work; it has notable resistance to polymyxin, with MICs for PMB and CST of 96 and 128 mg/L, respectively. A novel MFS-type tripartite efflux system, named CprABC, was identified in the genome of *Chryseobacterium* sp. PL22-22A. Heterologous expression and EPI assays indicated that the CprABC system is responsible for the polymyxin resistance of *Chryseobacterium* sp. PL22-22A. Structural modeling suggested that this efflux system provides a continuous conduit that runs from the CprB funnel through the CprC porin domain to pump polymyxins out of the cell. A specific *C*-terminal α-helix, CprBc, has an activation function on polymyxin excretion by CprB. The flavonoid compound baicalin was found to affect the allostery of CprB and/or obstruct the substrate conduit, and thus to inhibit extracellular polymyxin transport by CprABC.

**Conclusion:**

Novel MFS-type tripartite efflux system CprABC in *Chryseobacterium* sp. PL22-22A mediates resistance to polymyxins, and baicalin is a promising EPI.

## Introduction

Polymyxins, which have strong bactericidal activity against a range of Gram-negative bacteria, are a class of cyclic polypeptide antibiotics derived from fermentation products of the bacterium *Paenibacillus polymyxa* (Cai et al., 2015). Five different polymyxins (A, B, C, D, and E) were originally isolated, but only polymyxin B (PMB) and polymyxin E (colistin, CST) are available for clinical use for the treatment of Gram-negative bacterial infections (Poirel et al., 2017). Colistin differs from polymyxin B in the amino acid composition (D-phenylalanine at position 6 of polymyxin B is replaced by D-leucine in colistin) (Kwa et al., 2007). Polymyxins bind to the outer membrane (OM) lipopolysaccharide (LPS) of Gram-negative bacteria through the interaction of their cationic residues with the phosphate groups of lipid A, competitively displacing the Ca^2+^ and Mg^2+^ cations that link adjacent LPS molecules and then inserting their hydrophobic portions into the phospholipid layer, changing the permeability of the cell envelope, leading to leakage of cell contents and bacterial death (Velkov et al., 2013; Karaiskos et al., 2017).

Antibiotic resistance has emerged as a major threat to public health (Li et al., 2015). Multidrug-resistant Gram-negative bacteria (e.g., Enterobacteriaceae, *Acinetobacter*, and *Pseudomonas*) constitute a large proportion of resistant threats (Li et al., 2015). Polymyxins are one of a few remaining available active agents against carbapenem-resistant bacteria causing life-threatening infections in clinical settings and are increasingly being used as last-line therapeutic options against a number of multidrug resistant bacteria (Nation et al., 2014; El-Sayed Ahmed et al., 2020). These amphipathic lipopeptide molecules have been used for >50 years in both veterinary and human medicine (Silva et al., 2022). However, an increasing incidence of polymyxin-resistant bacteria, such as *Neisseria meningitidis*, *Proteus mirabilis*, and *Burkholderia* spp., has been reported in both nosocomial and community settings (Srinivas and Rivard, 2017).

The primary mechanism of polymyxin resistance in Gram-negative bacteria is modification of lipid A of LPS, which is a major component of the bacterial OM and the initial target of polymyxins (Nang et al., 2019). *eptA* and *arnBCADTEF* are responsible for modification of lipid A with positively-charged phosphoethanolamine and/or 4-amino-4-deoxy-L-arabinose (Olaitan et al., 2014), which results in decreased negative charge of the OM and hence decreased electrostatic interaction with polymyxins (Baron et al., 2016). Secondary polymyxin resistance mechanisms independent of modification of lipid A include the production of capsular polysaccharide, expression of efflux pumps, and increased expression of OM proteins. Major facilitator superfamily (MFS) transporters, which are among these resistance mechanisms, constitute the largest class of secondary transporters and share a similar folding topology and function (Huang et al., 2003; Jiang et al., 2013). Most MFS transporters are single-component (singlet, such as MdfA), but a few are tripartite efflux systems, for example EmrAB-TolC and EmrKY-TolC (Li et al., 2015).

In this study, we isolated a Gram-negative polymyxin-resistant strain, *Chryseobacterium* sp. PL22-22A, from aquaculture sewage. A novel MFS-type tripartite efflux system, encoded by genes *cprA*, *cprB*, and *cprC*, was identified in its genome. CprABC was proved to be a contributor to resistance to polymyxins. The flavonoid compound baicalin (BAC) showed efflux pump inhibitor activity by steric hindrance to prevent polymyxin transport by CprB.

## Materials and methods

### Isolation and identification of polymyxin resistant *Chryseobacterium* sp. PL22-22A

Aquaculture sewage samples were collected in Shandong Province, China, and spread onto Luria–Bertani (LB)-agar plates (0.5% yeast extract, 1% tryptone, 1% sodium chloride, 2% agar) containing 8 mg/L PMB (Sigma Co. Shanghai, China) and then incubated at 28°C for 24 h. All colonies with different phenotype on the plates were selected and cultivated three consecutive times on LB-agar with PMB to obtain single colonies. After further purification, one single colony, named PL22-22A, was selected and grown in pure culture, then 16 S rDNA was amplified by PCR and then sequenced to verify the purity. Antimicrobial susceptibility tests were performed to determine the minimum inhibitory concentrations (MICs) of nine antibiotics, including meropenem, cefixime, tetracycline, ciprofloxacin, florfenicol, amikacin, sulfamethoxazole, PMB, and CST, toward PL22-22A. Data were analyzed based on breakpoints defined by the Clinical and Laboratory Standards Institute (CLSI, 2020).

### Whole-genome sequencing, annotation, and analysis

The genome sequence of *Chryseobacterium* sp. PL22-22A was sequenced by using the Nanopore and BGISEQ-500 platforms at BGI Co., Ltd. (Wuhan, China). The PL22-22A genome was assembled by glimmer3^1^ with hidden Markov models (Tatusova et al., 2016). Genome annotation was performed using the Prokaryotic Genome Annotation Pipeline of NCBI.^2^ Genome alignment was performed by the PATRIC server (Brettin et al., 2015). A phylogenetic tree, including PL22-22A, was produced by using neighbor-joining algorithms in Molecular Evolutionary Genetics Analysis 7 (MEGA 7) software and 16S rDNA gene sequences (Jeong et al., 2016).

### Analysis of antibiotic resistance genes

Antibiotic resistance genes were analyzed by RAST and BLASTp based on the core dataset in the Antibiotic Resistance Genes Database (Liu and Pop, 2009). Multisequence comparison was carried out using Clustal Omega (Madeira et al., 2019) and ESPript (Robert and Gouet, 2014) software.

### Cloning of the tripartite transporter system-encoding *CprABC* genes

A fragment of the promoter sequence of the ampicillin-resistance gene (Pamp^r^) from pMD-18 T with a *Kpn*I site at the 5′-end was amplified using primers Pamp-F3 and Pamp-R3. The entire target gene *cprB* was amplified from genomic DNA of PL22-22A by PCR using primers CprB-F3 and CprB-R3. *cprA* and *cprC* with an *XbaI* site at the 3′-end were amplified by PCR using primers CprA-F3 and CprC-R3 from genomic DNA of PL22-22A. These fragments were fused to obtain fragment CdABC, which was digested with *XbaI* and *KpnI* and inserted into vector pMD18-T digested with *XbaI* and *KpnI*. The resulting recombinant plasmid pMD-CdABC was transformed into *Escherichia coli* DH5α, and this strain was named *E. coli* EcABC3. *E. coli* containing *cprB* (*E. coli* EcB1), *cprA* (*E. coli* EcA1), or *cprC* (*E. coli* EcC1), respectively, was also prepared. Combinations of two genes *cprB + cprA* (*E. coli* EcAB), *cprB* + *cprC* (*E. coli* EcBC), and *cprA* + *cprC* (*E. coli* EcAC) were constructed by using the same process as described above. *E. coli* DH5α and DH5α harboring pMD18-T vector (strain DT) were used as controls. Primer sequences are listed in Supplementary Table S1.

### Determination of MICs, MBCs and EC50 in the presence of efflux pump inhibitor

The MICs of PMB and CST for the various *E. coli* strains were determined in the presence of PMB and CST at 28°C for 24 h. MICs of PMB and CST in the presence of efflux pump inhibitors (EPIs) were also determined. Verapamil, carbonyl cyanide m-chlorophenyl hydrazone (CCCP), phenylalanine-arginine β-naphthylamide dihydrochloride (PAβN), reserpine (RES), and baicalin (BAC) were used as EPIs at final concentrations of 8.0, 0.1, 8.0, 8.0, and 8.0 mg/L, respectively. As control, MICs of BAC were also determined to check the viability of the bacteria as above. Trypan blue staining was operated when bacteria grow into OD_600_ = 0.8 and microscope direct counting method used to evaluate live/dead ratio. This experiment was performed three times independently. An aliquot of the initial inoculum for the MIC plate was similarly processed. The cells were resuspended in fresh media, plated onto LB, and the colonies enumerated after incubating for 24 h at 37°C. The MBC is defined as the first drug dilution which resulted in a 99.9% decrease from the initial bacterial titer of the starting inoculum or no growth. MBC/MIC ratio with tolerance defined by a ratio ≥ 32.

EC_50_ values of PMB and CST were determined according to growth inhibition. Briefly, 10 μL of bacterial suspension was added to 5 mL LB medium containing diluted concentrations of PMB and CST. OD_600_ values of the tested suspensions were measured when the control suspensions increased to OD_600_ = 1.0. The log of percentage inhibition based on OD_600_ values were regressed on the log of compound concentrations, and EC_50_ values were calculated. This experiment was performed three times independently. To assess the effect of EPIs on bacterial growth, 1 mL samples were taken from each bottle at selected time intervals (0, 1, 2, 3, 4, 6, 8, 10, 12, 14, 16, 20 and 24 h after the start of the experiment) and serial 10-fold dilutions were plated in triplicate onto LB agar plates. The numbers of cfu were counted after incubation for 24 h at 37°C.

### Molecular docking of CprB with polymyxins and baicalin

Transmembrane (TM) regions of proteins were identified using the TMHMM 2.0 server.^3^ Signal peptides were predicted using SignalP-6.0.^4^ Protein homology modeling and molecular docking analyses were performed as previously described (Zong et al., 2020). The homology model constructed for the selected MFS transporter was analyzed using Rosetta software (Leman et al., 2020) and Discovery Studio (Biovia, 2017) by using EmrAB. Hydrophobic surface features were analyzed using Discovery Studio (Biovia, 2017). The structure of polymyxins, for application as ligands, was obtained from Chemspider.^5^ The binding of CprB with PMB and CST was modeled using the CDOCKER protocol of Discovery Studio 2.0 (Biovia, 2017). Chemical bonds between CprB and polymyxins were demonstrated by 3D and 2D methods. Allosteric sites of CprB were analyzed by using AlloSite 2.0 (Huang et al., 2013).

## Results

### Multidrug-resistant *Chryseobacterium* sp. PL22-22A has notable resistance to polymyxins

Strain PL22-22A was isolated from aquaculture sewage under selection with 8 mg/L PMB. MIC analysis of different kinds of antibiotic, including a series of β-lactam, fluoroquinolone, tetracycline, chloramphenicol, aminoglycoside, sulfonamide, and glycopeptide antibiotics, revealed that PL22-22A is a multidrug-resistant strain (Supplementary Table S2). Interestingly, strain PL22-22A showed notable resistance to PMB (MIC 96 mg/L) and CST (MIC 128 mg/L).

**Figure 1:**
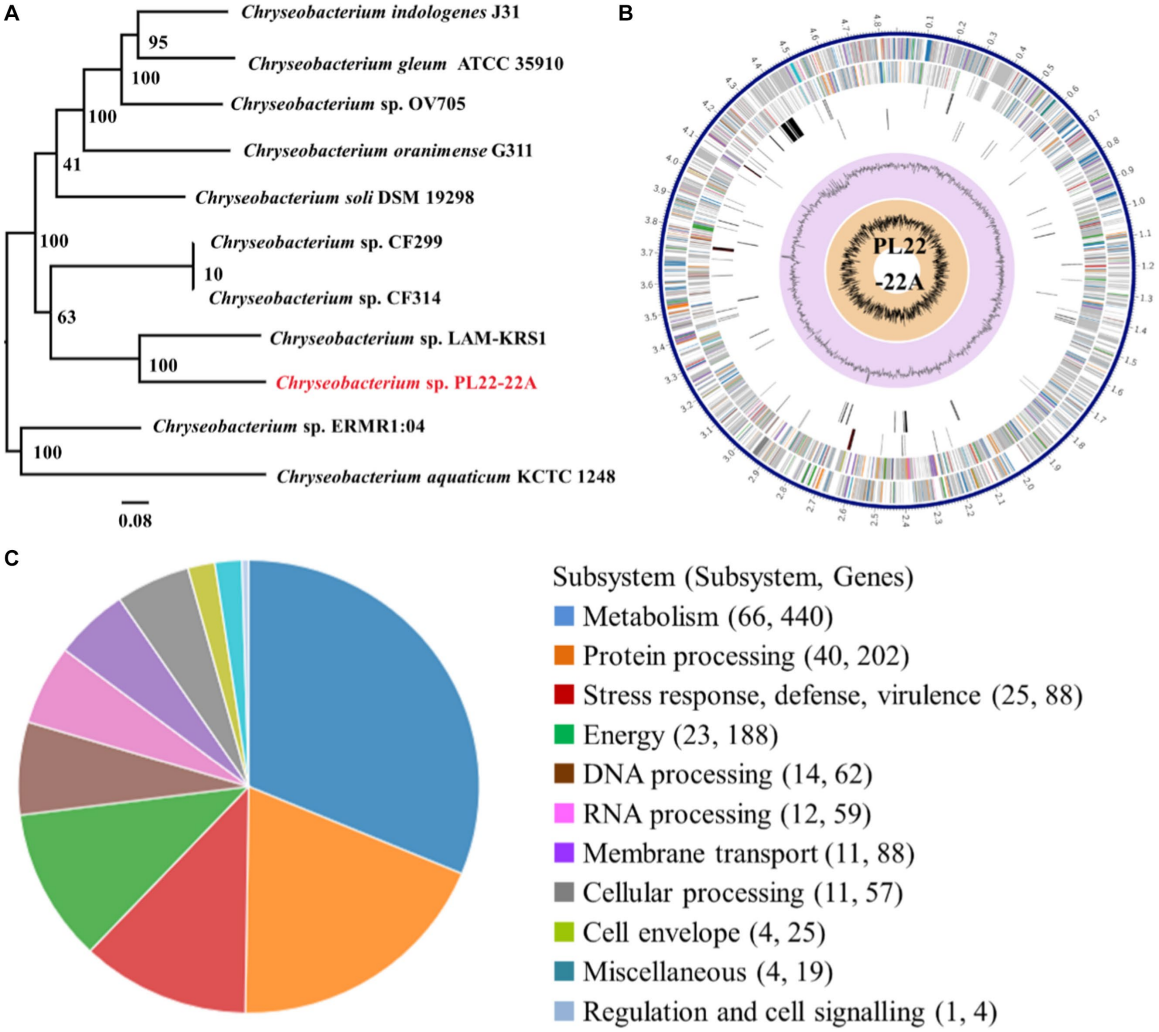
Identification and genomic characteristics of strain PL22-22A. **(A)** Neighbor-joining phylogenetic tree generated on the basis of 16S rDNA gene sequences. **(B)** PATRIC annotation of the genome of *Chryseobacterium* sp. PL22-22A. **(C)** Subsystem analysis of the genome of *Chryseobacterium* sp. PL22-22A.

Based on phylogeny of 16S rDNA genes, PL22-22A was identified as *Chryseobacterium* sp. (Figure 1A), which is an opportunistic pathogen. *Chryseobacterium* sp. PL22-22A harbors a 4,858,345 bp chromosome with 36.12 mol% G + C content (Figure 1B). The complete genome sequence was annotated; 857 functional proteins were assigned Enzyme Commission numbers, and 746 were assigned Gene Ontology terms (Supplementary Table S3). Subsystem analysis of PATRIC annotations indicated that strain PL22-22A contained 88 stress response-, defense-, and virulence-related genes, and 88 membrane transport-related genes (Figure 1C).

To investigate the mechanisms of multidrug resistance, antimicrobial resistance genes were identified in the genome. Genes that are responsible for resistance to β-lactams and tetracycline, including those encoding β-lactamase, metallo-hydrolase, and TetX, were identified (Supplementary Table S4). In addition, a considerable number of efflux pump-related genes were identified; MFS and ATP-binding cassette transporters were prominent classes, with 48 and 57 related genes (Supplementary Table S4), respectively. However, genes that were obviously responsible for polymyxin resistance, such as the lipopolysaccharide modification gene *pmrK*, which was reported to be the main mechanism of polymyxin resistance in Gram-negative bacteria (Moffatt et al., 2019), were not found in the genome.

### MFS tripartite transporter system CprABC contributes to resistance to polymyxins

In the *Chryseobacterium* sp. PL22-22A chromosome, the only three tripartite MFS efflux system genes, including PFY10_00045 (encoding a TolC family protein), PFY10_00050 (encoding a membrane fusion protein), and PFY10_00055 (encoding an inner membrane protein), were identified in a single operon. The components of this system PFY10_00055, PFY10_00050, and PFY10_00045, showed low identities (21.16–26.97%) with currently known MFS tripartite transporters, such as the three-component multidrug MFS-type efflux pumps EmrAB-TolC and EmrKY-TolC in *E. coli* (Supplementary Figures S1–S3). These *cpr* (Chryseobacterium Polymyxins Resistance) proteins in *Chryseobacterium* sp. PL22-22A were named CprABC.

**Figure 2:**
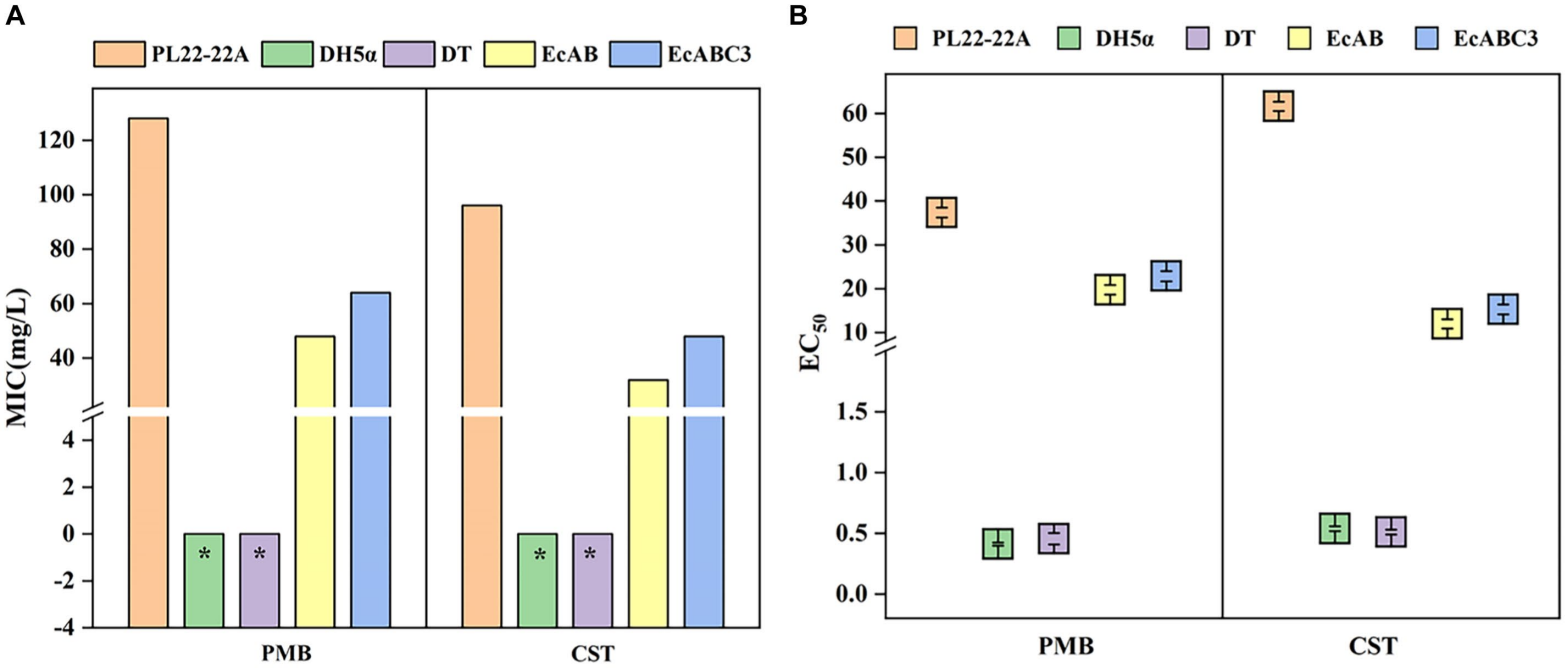
Minimum inhibitory concentrations (MICs) and EC_50_ of polymyxins toward different strains. **(A)** MICs of polymyxins toward different strains; **(B)** EC_50_ of polymyxins toward different strains. DH5α, *Escherichia coli* DH5α (control); DT, *E. coli* DH5α with pMD-18 T (control); EcAB, *E. coli* DH5α expressing *cprA-cprB*; EcABC3, *E. coli* DH5α expressing *cprABC*; PMB, polymyxin B; CST, colistin. *MIC<2 mg/L.

Potential functions of *Chryseobacterium* sp. PL22-22A CprABC in multidrug resistance were analyzed by expression of the genes in *E. coli*. MICs of nine antibiotics toward *E. coli* EcABC3 (containing *cprA*, *cprB*, and *cprA*) and EcAB (containing *cprA* and *cprB*) were tested. Compared with the control strains DH5α and DT (MICs <2 mg/L), EcABC3 (containing all three CprAB-TolC-encoding genes) gained resistance to PMB and CST (MICs 64 mg/L and 48 mg/L, respectively) (Figure 2A). This strain showed no resistance to the other seven tested antibiotics (meropenem, cefixime, tetracycline, ciprofloxacin, florfenicol, amikacin and sulfamethoxazole) (Supplementary Table S2). These data indicate that the CprABC-encoding genes confer polymyxin-specific resistance.

Expression of the genes for incomplete transporter systems (i.e., CprAB of CprABC) gave different results. In the EcAB-expressing *E. coli* strain, PMB resistance (MIC 48 mg/L) and CST resistance (MIC 32 mg/L) increased compared with those of control strains DH5α and DT. Thus, in the presence of *cprB*, *E. coli* can gain resistance to PMB and CST.

The EC_50_ of PMB and CST were significantly lower for *E. coli* DH5α (0.411 ± 0.013 and 0.534 ± 0.02 mg/L respectively) and DH5α with pMD-18 T (0.455 ± 0.047 and 0.509 ± 0.02 mg/L respectively), compared with *Chryseobacterium* sp. PL22-22A (37.35 ± 1.102 mg/L and 61.61 ± 1.053 mg/L respectively), indicating that a lower PMB and CST concentrations are needed in DH5α to reach 50% of the maximum effect. While, after expressing *cprA*, *cprB* or *cprABC* in *E. coli*, the EC_50_ of PMB were significantly raised to 19.74 ± 1.103 and 22.85 ± 1.153, the EC50 of CST raised to 11.99 ± 1.063 and 15.29 ± 1.127 (Figure 2B). EC_50_/MIC showed the consistent results (Supplementary Table S5). This confirmed that MFS tripartite transporter system CprABC contributes to resistance to polymyxins.

### Structure of each component of the tripartite transporter system CprABC

**Figure 3:**
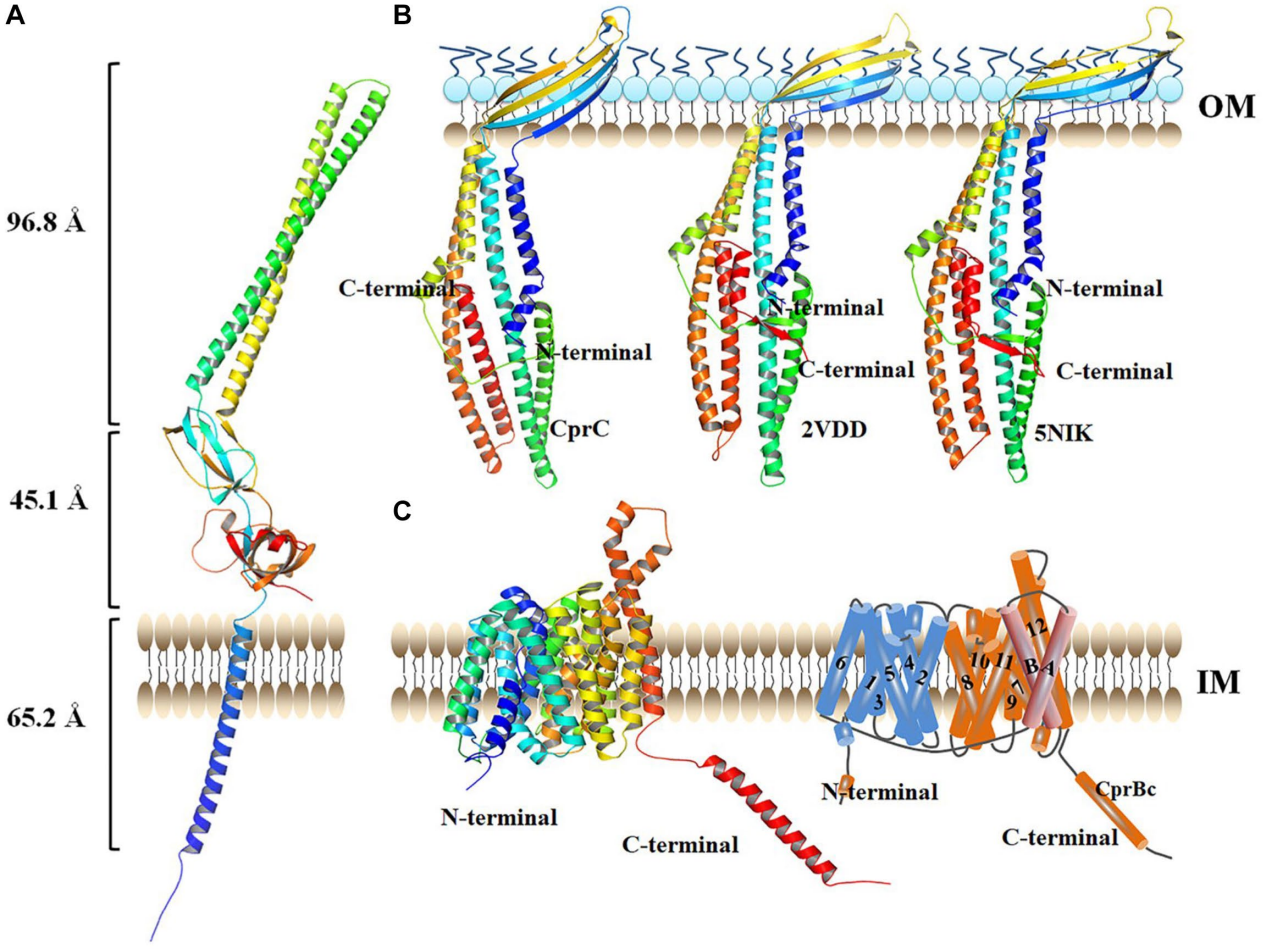
Structure of each monomer in the tripartite transporter complex CprABC from *Chryseobacterium* sp. PL22-22A. **(A)** Structure of CprA monomer. **(B)** Structure of CprC monomer. **(C)** Structure of CprB monomer, left: structure viewed in the plane of the membrane; right: topology diagram. IM, inner membrane; OM, outer membrane. AcrAB-TolC (PDB: 2VDD) was used as homology model.

CprA is a membrane fusion protein of 370 amino acid residues and shorter than EmrA and EmrK (390 and 387 amino acid residues, respectively) (Supplementary Figure S1). CprA showed 26.97 and 24.56% sequence identity with EmrA and EmrK, form from *E. coli, respectively.* Similarly to EmrA, CprA has an *N*-terminal cytoplasmic domain preceding a TM helix (Figure 3A). The *N*-terminal TM helix (Hf) is directly attached to the β-barrel domain in CprA. This helix may lead to localization in the cell membrane and assembly as an inner membrane (IM) component of the tripartite MFS system.

CprC of CprABC in *Chryseobacterium* sp. PL22-22A is a 370-amino acid protein. Compared with TolC in the tripartite multidrug efflux proteins AcrAB-TolC (PDB: 2VDD) and MacAB-TolC (PDB: 5NIK), CprC is shorter at the *C*-terminus (Supplementary Figure S1). The shortened *C*-terminal fragment of CprC was constituted by a minor α-helix and a β-sheet, compared with the equatorial domains of TolCs in AcrAB-TolC and MacAB-TolC (Figure 3B). The CprC monomer projects across the intermembrane periplasmic space and is anchored in the OM by a contiguous barrel domain (Figure 3B).

The IM component of the tripartite MFS system in *Chryseobacterium* sp. PL22-22A, CprB, consists of 530 amino acid residues; it is thus larger than the tripartite MFS system IM proteins EmrB and EmrY (both 512 amino acid residues). Additional sequence, which is absent from EmrB and EmrY, is located at the *C*-terminus of CprB (from G489-L530, this sequence is named CprBc hereafter) (Supplementary Figure S3). Homology modeling showed that CprB contains 14 putative TM helices, of which helices H1–H12 form two domains (the *C*-domain and *N*-domain) that were observed previously for MFS transporters such as PepTSo (Newstead et al., 2011) and EmrB (Yousefian et al., 2021) (Figure 3C, left). As in previous MFS transporter structures, the *N*- and *C*-terminal six-helix bundles, formed by helices H1–H6 and H7–H12 respectively, come together to form a “V”-shaped transporter, related by a pseudo two-fold symmetry axis running perpendicular to the membrane plane. Similarly to PepTSo, CprB has two additional TM helices, HA and HB, which are inserted into the cytoplasmic loop connecting the *N*- and *C*-terminal bundles (Figure 3C, right).

### Overall structure of the tripartite transporter system CprABC

**Figure 4:**
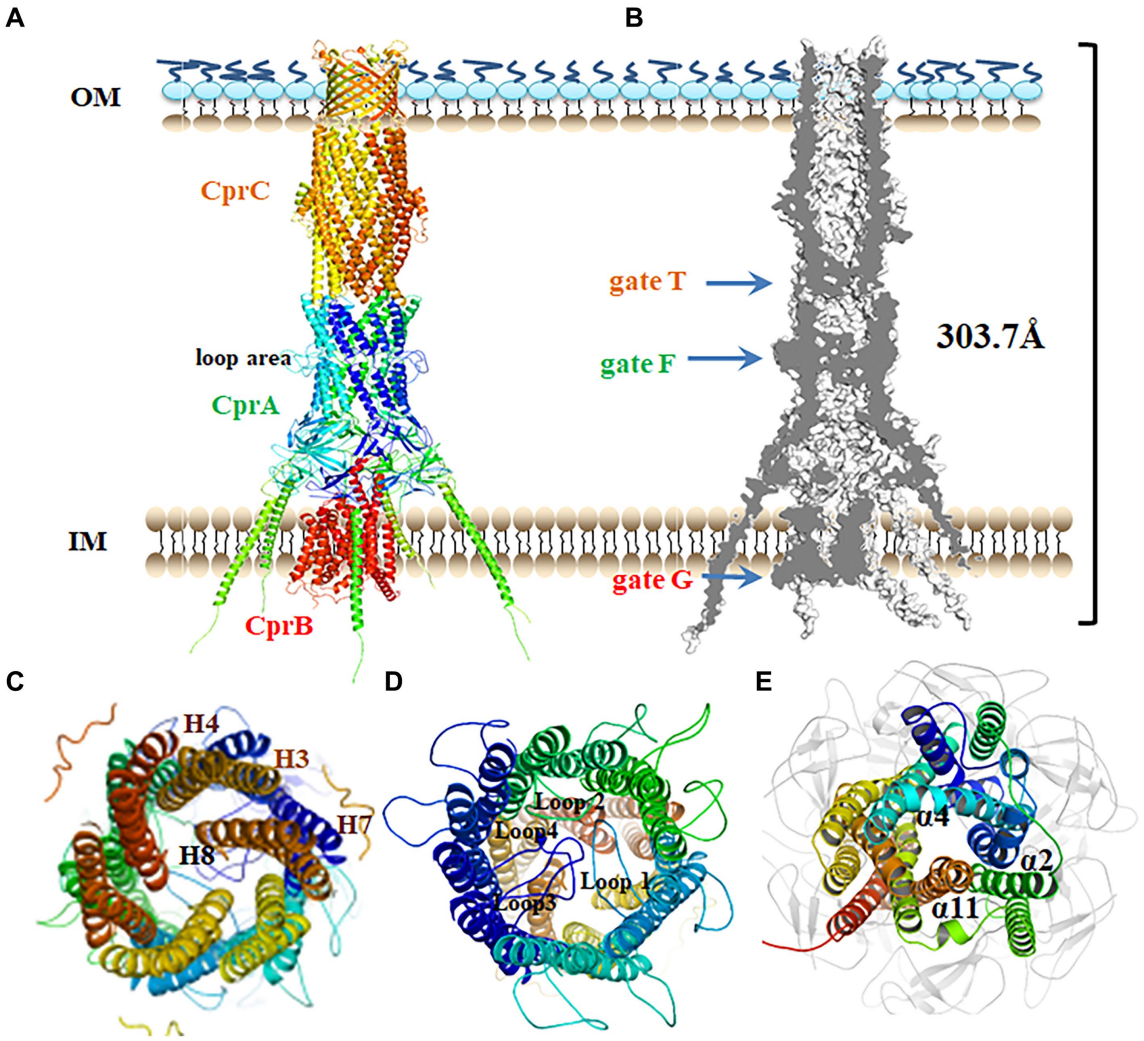
Putative assembly of the CprABC pump. **(A)** Overall structure of the CprABC pump seen from the periplasmic side. **(B)** Cross-section through CprABC. **(C–E)** Gates T, F and G seen from the OM side. IM, inner membrane; OM, outer membrane.

In Gram-negative bacteria, tripartite efflux pumps span both the inner and outer membranes, providing a continuous seal for drugs to bypass the periplasm. To analyze the details of polymyxin efflux mediated by CprABC, this tripartite transporter system was modeled and then assembled by using SWISS-MODEL and Discovery Studio software. Based on EmrAB from *E. coli* (Tanabe et al., 2009), membrane fusion component CprB is modeled as a homohexamer, CprC as a typical homotrimer, and IM component CprB as an MFS-type monomer (Figure 4A). CprABC is a 303.7-Å long transporter complex. A slice through the model shows the continuous conduit that runs from the CprB funnel through the CprB porin domain (Figure 4B). In this conduit, there were three conspicuous domains, one in each component of CprABC, crossing the channel-like gates; we named these gates G, F, and T, respectively (Figure 4B).

In this model, CprC has a typical closed state of the entrance, which is constituted by 12 tunnel-forming α-helices. This entrance must be opened for substrates to access the exit duct (Andersen et al., 2002). Similar to TolC in *E. coli* (Andersen et al., 2002), three inner coiled coils (comprising helices H7 and H8), and also gate T in CprABC, are proposed to be allosteric for tunnel opening (Figure 4C). Because of the homologous polymerization, homohexamer CprA presents a loop region in the middle-upper part (Figure 4D), which is different compared with the model of the CprA monomer. Gate F in CprA is composed of four inner loops (loops 1–4) located in that loop region. Helices α2, α4, and α11 in CprB constitute a narrow entrance, gate G (Figure 4E), and may be essential for the transition between the inward and outward conformations of the MFS-type transporter (Jiang et al., 2013).

### The CprBc fragment affects the function of CprABC

**Figure 5:**
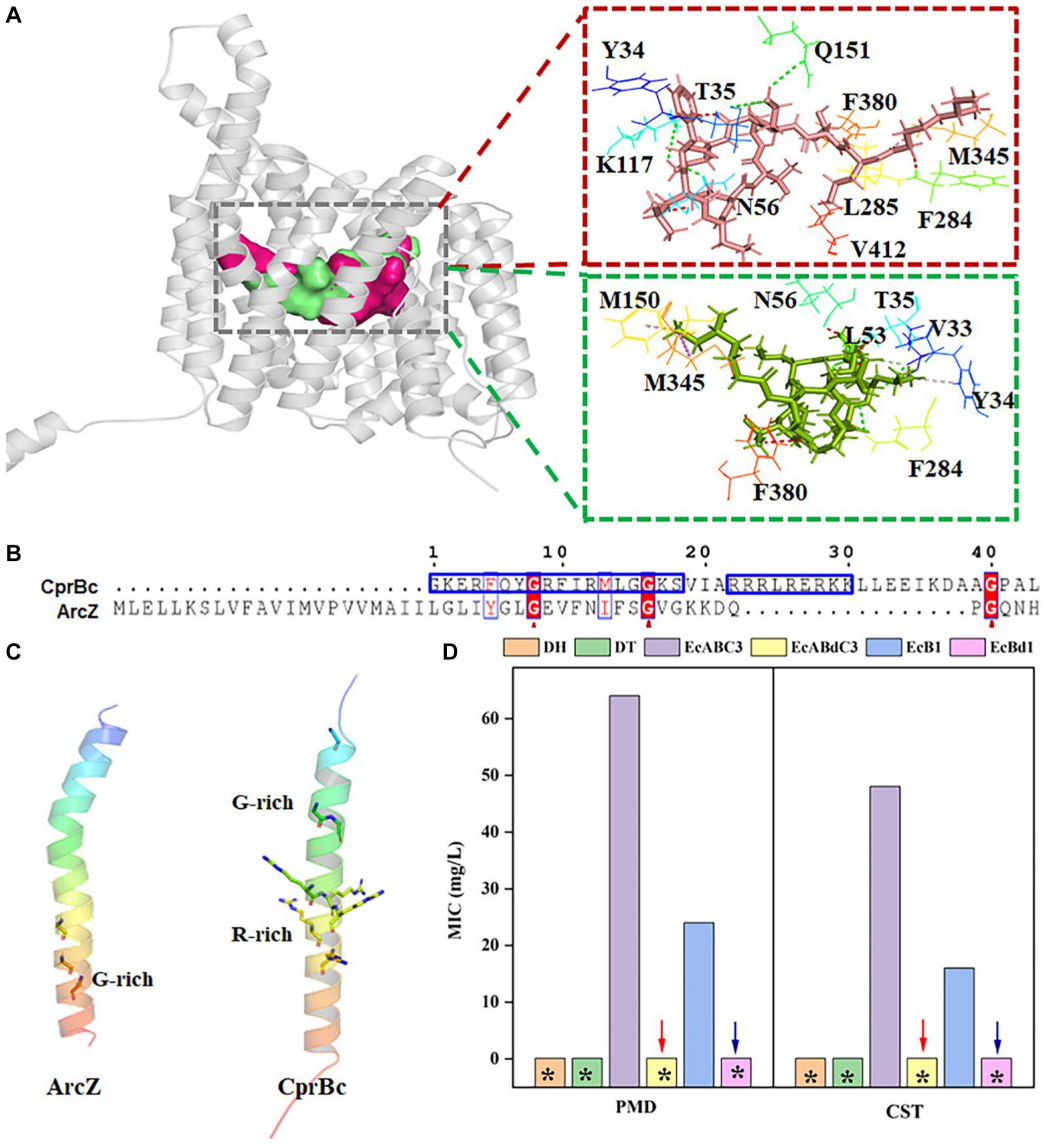
The *C*-terminal fragment CprBc of CprB contributes to activation of the CprABC complex. **(A)** Predicted PMB- and CST-binding sites and binding amino acids in CprB. PMB and CST are shown as red and green sticks, respectively; PMB- and CST-binding sites are shown by red and green surfaces; PMB- and CST-binding amino acids are shown by lines. **(B)** Alignment of amino acid sequences of CprBc and ArcZ. Red arrows indicate conserved glycine sites. **(C)** Structural comparison of CprBc and ArcZ (from *E. coli*, PDB: 5nc5). **(D)** Influence of CprBc deletion on CprABC pumping of polymyxins. DH, *E. coli* DH5α (control strain); DT, *E. coli* DH5α with pMD-18 T (control strain); EcABC3, *E. coli* DH5α expressing *Chryseobacterium* sp. PL22-22A *cprABC*; EcABdC3, *E. coli* DH5α expressing *Chryseobacterium* sp. PL22-22A *cprA*, *cprC*, and *cprBd* (truncated CprB with CprBc deleted); EcB1, *E. coli* DH5α expressing *Chryseobacterium* sp. PL22-22A *cprB*; EcBd1, *E. coli* DH5α expressing *Chryseobacterium* sp. PL22-22A *cprBd* (truncated CprB with CprBc deleted). *MIC<2 mg/L.

In tripartite efflux systems such as EmrAB-TolC and EmrKY-TolC, IM components were a key factor in the efflux of free fatty acids (Li et al., 2015). In the CprABC tripartite efflux system, MFS-type IM proteins CprB has two adjacent binding sites that combine with PMB and CST, respectively, in its intermediary channel (Figure 5A), although their binding amino acids are different. Y34, T35, N56, and F284 seem to be essential amino acids for combination with PMB and CST (Figure 5A). Redundant sequence from G487 to L530, i.e., CprBc, forms a specific α-helix fragment of CprB. Its function was unknown. Moreover, we found that CprBc contains an arginine (R)-rich domain and a glycine (G)-rich domain, which is important for interaction between AcrZ and AcrB that in RND-type AcrAB-TolC complex (Newstead et al., 2011) (Figure 5B). The G-rich domain of CprBc is at its *N*-terminus, and the R-rich domain is in the middle; this contrasts with AcrZ, in which the G-rich domain is *C*-terminal, and there is no R-rich domain (Figure 5C). Considering that AcrZ enhanced the ability of the AcrAB-TolC pump to export certain classes of substrates (Hobbsa et al., 2012), we investigated the function of CprBc in substrate selectivity.

CprB was truncated by deletion of CprBc (M1-F487 were retained) and this incomplete CprB was expressed in *E. coli*. EcAB_d_C3 (containing the CprABC-encoding genes with truncated *cprB*) was sensitive to PMB and CST (MIC <2 mg/L). This sensitivity was the same as that of the control strains *E. coli* DH5α and DT. In comparison, EcABC3, which contains the complete CprABC encoding genes, was resistant to PMB and CST (MICs 64 and 48 mg/L, respectively) (Figure 5D). A similar phenomenon was observed when truncated *cprB* was expressed alone in strain EcB_d_1, compared with EcB1 (containing complete *cprB*) (Figure 5D). Loss of resistance to PMB and CST showed that CprBc activates CprB to excrete PMB and CST.

### Baicalin restores sensitivity to polymyxins as an inhibitor of allostery in CprB

**Figure 6:**
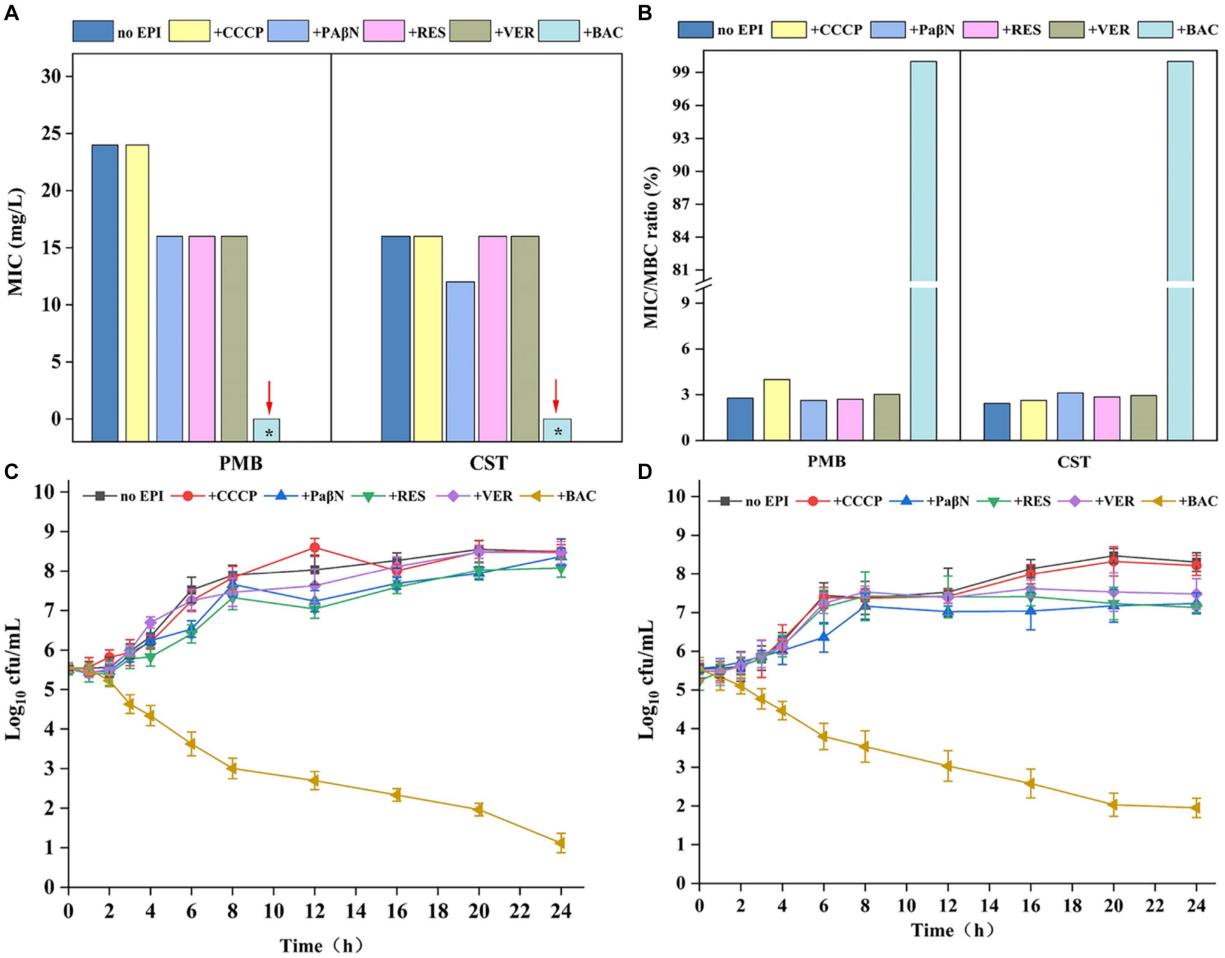
Baicalin decreasing resistance to polymyxins. **(A)** Effect of efflux pump inhibitors (EPIs) on the MIC of strain EcB1. **(B)** Effect of efflux pump inhibitors (EPIs) on the MBC of strain EcB1; **(C)** Growth curves of EcB1 in PMB containing medium in the presence of EPIs; **(D)** Growth curves of EcB1 in CST containing medium in the presence of EPIs. No EPI, strain EcB1 without EPIs; +, with different EPIs, i.e., CCCP, carbonyl cyanide (*m*-chlorophenyl)hydrazone; PaβN, phenylalanine-arginine β-naphthylamide dihydrochloride; RES, reserpine; VER, verapamil; BAC, baicalin. *MIC<2 mg/L.

EPIs are molecules that inhibit efflux pumps by one or more mechanisms, leading to inactive drug transport (Sharma et al., 2019). MIC analysis showed that the typical EPIs verapamil, CCCP, PaβN, and reserpine had no or only slight decreasing effects on PMB and CST resistance in EcG1 strain. The MICs toward PMB and CST showed no significant difference in the presence and absence of CCCP (24.0 mg/L). Although verapamil, PaβN, and reserpine decreased the MIC of PMB from 24.0 mg/L to 16.0 mg/L, strain EcB1 retained resistance to polymyxins (Figure 6A). BAC (CAS: 21967-41-9) is a potential flavonoid EPI screened from a Traditional Chinese Medicine Active Compound Library that was proven to be an MFS transporter EPI in *Brevundimonas* sp. M20 (unpublished data). Here, the effect of BAC on CprB was investigated. At the concentration of 8 mg/L, BAC showed no bacteriostatic activity (Supplementary Figure S4) for strain EcG1, while, it decreased the MIC of PMB and CST to <2.0 mg/L for both drugs (Figure 6A). MBC result also showed a significant reduction in the present of BAC (<2 mg/L) and BAC restored the sensitivity for PMB and CST. While other traditional EPIs showed no equivalent effects (Figure 6B) and the strain maintained tolerance. The minimum inhibitory/bactericidal concentration (MIC/MBC) ratio for polymyxins confirmed that.

Log-linear growth rates in the EPIs-free control showed no significant difference for EcB1 strain in the traditional EPIs (CCCP, verapamil, PaβN, and reserpine) as determined. While, in the presence of BAC, cfu of EcG1 for PMB (Figure 6C) and CET (Figure 6D) were significantly reduced. Considering low doses of BAC (8 mg/L) has no bacteriostasis but could deduce MIC for more than 8-fold (Figure 6A) at this concentration. These results indicated that polymyxins and BAC reveal a synergistic effect in terms of bacterial resistance.

### BAC obstruct the passage of polymyxins through the MFS transporters pump

**Figure 7:**
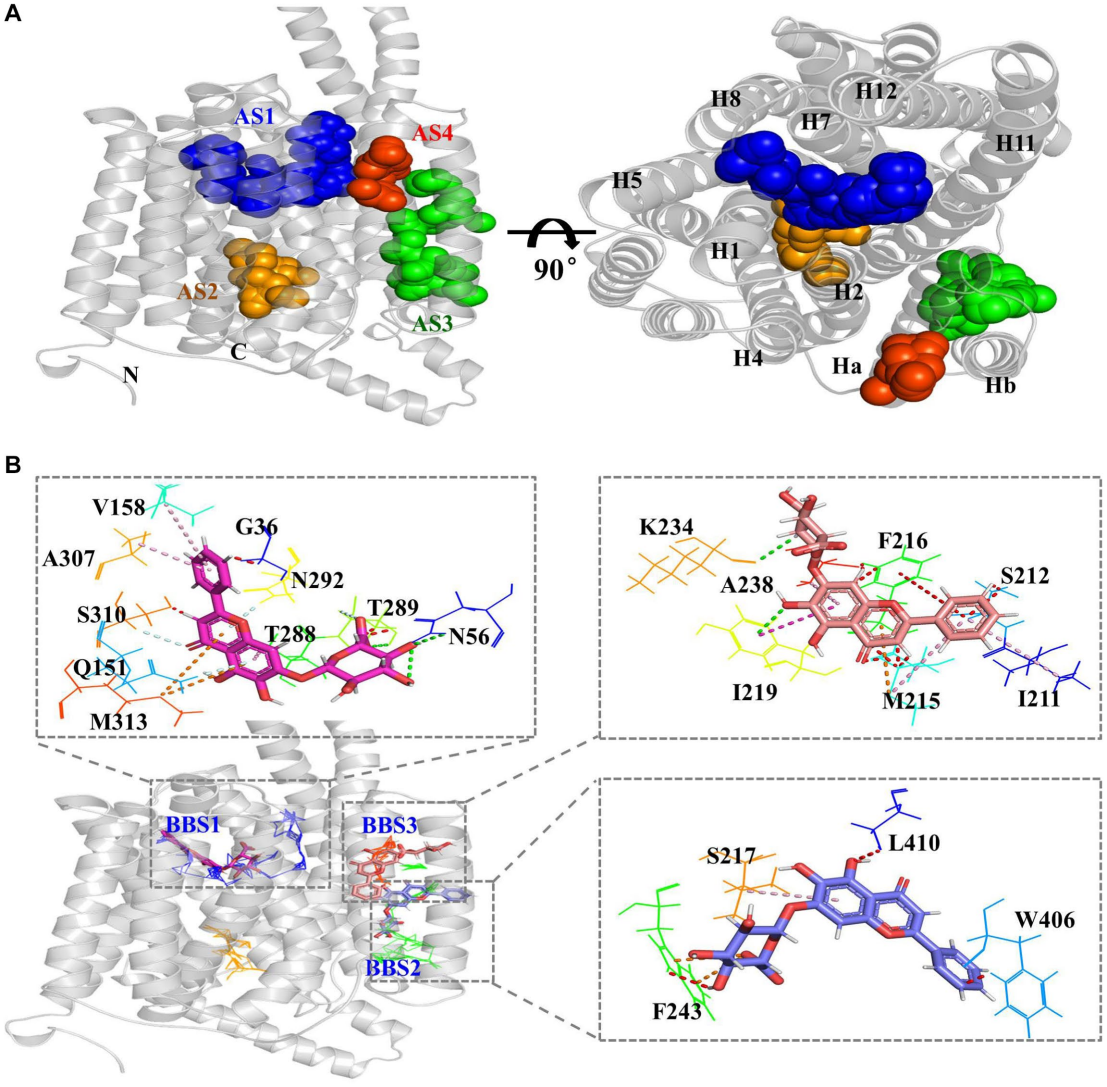
BAC obstruct the passage of polymyxins through the MFS transporters pump. **(A)** Predicted allosteric sites (AS) of CprB. AS1–AS4 are represented in blue, orange, green, and red, respectively; N, *N*-terminus; C, *C*-terminus. **(B)** Predicted BAC-binding sites (BBS) and binding amino acids in *Chryseobacterium* sp. PL22-22A CprB. AS1–AS4 are shown by blue, orange, green, and red lines, respectively; BAC in BBS 1–3 is shown by red, orange, and violet sticks, respectively.

MFS transporters pump their substrates based on conformational changes between the outward and inward conformations (Reddy et al., 2012; Yan, 2015). According to analysis using AlloSite software, CprB was predicted to have four allosteric sites (AS1–AS4) in the protein (Figure 7A). AS1 was located at the top of the central TM channel of CprB (adjacent to the periplasmic space) (Figure 7A, blue). AS2 was predicted to be adjacent to cytoplasmic side and was beneath AS1 (Figure 7A, orange). AS1 and AS2 were surrounded by the channel-forming helices H1, H2, H4, H5, H7, H8, H11, and H12. AS3 and AS4 were located outside of the substrate channel (Figure 7A, green and red), between Ha and Hb (Figure 7A), whose function is still unclear in 14-TM-helix-containing MFS transporters (Newstead et al., 2011). This result indicated a possibility of allosteric functions of Ha and Hb in the novel MFS-type pump CprB.

To investigate the mechanism of inhibition of transport by flavonoid compound BAC, it was docked with CprB. Three BAC-binding sites (BBSs) were predicted in CprB, near AS1, AS3, and AS4, respectively (Figure 7B). It was predicted that ten, seven, and four amino residues bind with BAC in BBS1, BBS2, and BBS3, respectively. Q151 and N56 in BBS1 site, with are also in AS1 site, may play a crucial role. Most of the binding was by hydrophobic amino residues via π-alkyl and unfavorable bump interactions (Figure 7B). Thus, BAC may act as an allosteric inhibitor-type EPI. Furthermore, BBS1 is located in the central TM channel (Figure 7B); PMB and CST must pass this when they are pumped-out. Thus, in addition to allosteric inhibition, BAC appears to directly obstruct the passage of substrate through the transporter. This competitive effect caused a dysfunction for CprABC pump out polymyxins and resulted in a polymyxins sensitive.

## Discussion

The increase in microbial antibiotic resistance is a major worldwide health problem. Severe infections caused by multidrug-resistant bacteria, as well as the lack of new antibiotics against Gram-negative pathogens, have led to reconsideration of old antibiotics (Baron et al., 2016). Polymyxin antibiotics are increasingly being used as last-line therapeutic options against a number of multidrug-resistant bacteria (Moffatt et al., 2019). Worryingly, the identification of polymyxin-resistant bacteria has been documented with increasing frequency worldwide (Rodriguez-Santiago et al., 2021). *Chryseobacterium* sp. PL22-22A, isolated in this study, belongs to the species *Chryseobacterium*, which can cause infections in humans (Sharma et al., 2016). Here, *Chryseobacterium* sp. PL22-22A was proved to be a multidrug-resistant bacterium with prominent resistance to polymyxins B and E (i.e., CST).

Generally, the mechanism of resistance to polymyxins is alteration of LPS structure and charge, such as by addition of 4-amino-L-arabinose, phosphoethanolamine, and/or galactosamine (Moffatt et al., 2019). However, efflux systems were also proved to be involved in lower susceptibility of bacteria to polymyxins (Rodriguez-Santiago et al., 2021). Active efflux decreases the intracellular concentration of substrate compounds (i.e., drugs) to subtherapeutic levels, thereby directly conferring resistance. Efflux pumps such as MdtEF in *E. coli* (Liu et al., 2020); AcrAB–TolC (Padilla et al., 2010), KexD (Sun et al., 2020), and KpnEF (Srinivasan and Rajamohan, 2013) in *Klebsiella pneumoniae* were related to changes in susceptibility to polymyxins. However, MFS-type transporters have rarely been verified to be involved in polymyxin resistance. Here, a tripartite MFS-type efflux system, CprABC, was identified in *Chryseobacterium* sp. PL22-22A. To our knowledge, this is the third MFS-type tripartite efflux system after EmrAB-TolC (Yousefian et al., 2021) and EmrKY-TolC (Li et al., 2015; Pasqua et al., 2019). While, gene of this MFS-type tripartite transporter system is conserved in Chryseobacterium based on Blast of Genebank (date not shown). For now, only 26 strains could identified this CprABC type tripartite transporter system genes and maximum identity is 81.71% from *Chryseobacterium* sp. CJ51. In other gram negative bacteria, no CprABC type tripartite transporter system gene was identified. It seems that CprABC is a Chryseobacterium-specific tripartite transporter system.

CprAB present polymyxins resistance as similar extent as CprABC when expressed in *E. coli* (Figures 1, 5). This result shows the CprC-independent function of CprAB, which is like the TolC-independent function of another MSF type efflux transporters EmrAB in resistance to fatty acid salts in *Salmonella enterica* (Yoneda et al., 2022). While, for other tripartite drug efflux systems, outer membrane protein of *E. coli* such as TolC (be necessary for the function of AcrAB) and OprM (be necessary for the function of MexAB), also could be utilized by MexXY as the outer membrane component (Mine et al., 1999). Thus, as a MFS type tripartite efflux system, CprAB seems to be outer membrane channel nonspecific and may use TolC (CprC-like protein) to form a functional tripartite efflux pump in expressed *E. coli*. Further investigations on the necessary of outer membrane protein for the function of CprAB are needed. Additionally, the vast majority of MFS pumps are single-component or “singlet” pumps in the IM (Tal and Schuldiner, 2009). They take up the drug from the cytosol and the periplasm and function with porins or other types of protein channel to make the efflux process effective (Li et al., 2015). Tripartite MFS transporters seem to be anomaly in this family. Similar to RND transporters (Li et al., 2015), these MFS members are located in the IM but must interact with a periplasmic adaptor protein and an OM channel, thus producing a tripartite complex spanning the IM, the periplasm, and the OM. These multicomponent exporters can capture drugs from the periplasm and the IM, and directly excrete them from the cell, so that the re-entry of the drug requires the slow traversal of the OM (Nikaido, 2003). The competition between the influx and efflux processes ultimately determines the steady state of the drug molecule in the bacterial cell (Li et al., 2015).

In *E. coli*, the typical tripartite complex AcrAB-TolC (spanning the IM to the OM) was proved to be affected by a 49-amino acid protein, AcrZ. AcrZ interacts with the TM portion of the multidrug efflux pump AcrB and increases resistance of the bacterium to its antibiotic substrates (Hobbsa et al., 2012). This influence occurred when AcrZ bound to AcrB in lipid environments. AcrZ binding allosterically modulates AcrB activity by changing the conformation of the drug-binding pocket (Du et al., 2020). In the *Chryseobacterium* sp. PL22-22A CprABC complex, we found that a short *C*-terminal fragment of CprB_cb_ has similar function to AcrZ. Deleting this fragment, CprBc, eliminated the polymyxin resistance of strain EcABdC3 and the efflux function of the CprABC complex (MICs of PMB and CST <2 mg/L). The function of the α-helix CprBc on CprB in excretion of PMB and CST requires further study.

Identification of EPIs is a promising strategy for fighting resistance to antibiotics (Kapp et al., 2018). It has been established that EPIs active against Gram-negative bacteria commonly meet these criteria: (i) they can cross the OM; (ii) they enhance the activity of antibiotics; (iii) they show no activity in mutants lacking the respective efflux pumps; (iv) they decrease extrusion and increase accumulation of efflux pump substrates; and (v) they do not affect the proton gradient across the cytoplasmic membrane (Lamut et al., 2019). In essence, the function of a pump depends on conformational alterations (Li et al., 2015). Thus, allosteric inhibitors are effective in inactivating pumps. EPIs such as pyridopyrimidines and pyranopyridines bind to a hydrophobic trap of AcrB, halt the catalytic efflux cycle and likely also compete with drug binding (Wang et al., 2017). Theoretically, proton conductor type EPIs, such as CCCP (a proton uncoupler), can restore the sensitivity of bacteria to a variety of antibiotics by abolishing the energy source that from the proton motive force (Eicher et al., 2014). Most MFS transporters are monocomponent and the proton translocation residues are mainly located on the membrane-embedded surface of the C-terminal helices and separated from the substrate translocation pathway (Tamura, 2003). While, tripartite MFS, such as EmrAB-TolC, is intrinsic resistance to CCCP. In this study, we confirmed that the typical EPIs verapamil, CCCP, PaβN, and reserpine had no or only a slight decreasing effect on PMB and CST resistance mediated by the CprB. Thus, further investigations on this intrinsic resistance to EPIs will be a valuable subject.

However, the flavonoid BAC eliminated the resistance of cells expressing CprB to polymyxins. Structural simulations indicated that BAC is an allosteric inhibitor that binds to CprB at three binding sites near allosteric sites of CprB. Thus, this BAC approach that obstructing substrate translocation pathway is different from the traditional proton translocation method (such as CCCP). Further investigations and confirmations, such as EtBr accumulation experiments, druggability of BAC, drug safety evaluation based on animal experiment, are essential to evaluate the mechanism of this inhibitor.

## Conclusion

The multidrug-resistant bacterium *Chryseobacterium* sp. PL22-22A is resistant to polymyxins. In this strain, the IM MFS transporter CprB forms part of a tripartite system with its periplasmic and OM partners CprA and CprC, and is responsible for the resistance to polymyxin. CprABC traverses the IM and OM, providing a continuous conduit that runs from the CprB funnel through the CprC porin domain to pump polymyxins out of the cell. The flavonoid BAC was found to be a potential EPI of CprABC by affecting allostery and/or obstructing substrate passage through CprB. Furthermore, α-helix CprBc may have an activation function in CprB-dependent excretion of polymyxins.

## Data availability statement

The complete sequence of the *Chryseobacterium* sp. PL22-22A genome was deposited in GenBank under accession no. CP115858 (https://www.ncbi.nlm.nih.gov/nuccore/CP115858.1).

## Author contributions

LZ: Investigation, Resources, Writing – original draft. MW: Investigation, Resources, Writing – original draft. RQ: Investigation, Writing – original draft. YY: Investigation, Writing – original draft. YL: Investigation, Writing – original draft. NR: Software, Writing – original draft. ZF: Software, Writing – original draft. QL: Formal analysis, Investigation, Writing – original draft. GC: Project administration, Writing – review & editing. GZ: Conceptualization, Funding acquisition, Supervision, Visualization, Writing – review & editing.

## Footnotes

^1^http://www.cbcb.umd.edu/software/glimmer/

^2^http://ncbi.nlm.nih.gov/genome/annotation_prok/

^3^https://services.healthtech.dtu.dk/service.php?TMHMM-2.0

^4^https://services.healthtech.dtu.dk/services/SignalP-6.0/

^5^http://www.chemspider.com/
