# Supplementary material for: A novel major facilitator superfamily-type tripartite efflux system CprABC mediates resistance to polymyxins in Chryseobacterium sp. PL22-22A
Source: Front Microbiol. 2024 Feb 23;15:1346340. doi: 10.3389/fmicb.2024.1346340 (PMC11002906; doi:10.3389/fmicb.2024.1346340)
Supplement: Supplementary file 1 [file Presentation_1.pdf]

Table S1. Primers sequence (5′→3′) used in this study

Primer ID	Primer sequences (5’-3’)
**For tripartite transporter system genes *emrFG-tolC* expression**	
Pamp-F3	GGTACCGACGAAAGGGCCTCGTGATAC
Pamp-R3	GCCCAGATCGGTATCGGTCATACTCTTCCTTTTTCAATATTATTGAAGC
CprB-F3	GCTTCAATAATATTGAAAAAGGAAGAGTATGTATAACAAAGGCTTATATCATGATTGG
CprB-R3	TCTAGATTAAAGTGTTCCCGTAGATTTTAAAAG
**For two-combined transporter system genes *emrFG-tolC* expression**	
CprA-R1	TCTAGAGACCAGCCGCATCTTTTATTTC
Pamp-F3, Pamp-R3, CprB-F3 and CprA-R1are presented above and used for *cprB- cprA* expression	

Table S2. MICs of antibiotics for different strains (mg/L)

	meropenem	Cef	Tet	Cip	Flo	Amk	PMB	Sul	CST	BAC
PL22-22A	56	>128	>128	8	64	>128	96	>128	128	>128
EcABC3 (containing *cprA*, *cprB* and *cprC*)	<2	<2	<2	<2	<2	<2	64	<2	48	>128
EcAB (containing *cprA* and *cprB*)	/	/	/	/	/	/	48	<2	32	>128
DH5α (Control)	<2	<2	<2	<2	<2	<2	<2	<2	<2	>128
DT (DH5α harboring pMD18-T)	<2	<2	<2	<2	<2	<2	<2	<2	<2	>128

Abbreviations: Mer: Meropenem; Cef: Cefixime; Tet: Tetracycline; Cip: Ciprofloxacin; Flo: Florfenicol; Amk: Amikacin; PMB: Polymyxin B; Sul: Sulfamethoxazole; CST: Colistin; BAC: baicalin; /: untested.

Table S3. General features of the *C. daecheongense* PL22-22A genome

Features	Genome
Total number of base pairs	4,858,345 bp
G+C content (%)	36.12
Total genes	4,451
Repeat Regions	58
tRNA	69
rRNA	10
Hypothetical proteins	1,915
Proteins with functional assignments	2,536
Proteins with EC number assignments	857
Proteins with GO assignments	746
Proteins with KEGG assignments	642
Proteins with PATRIC genus-specific family (PLfam) assignments	3,601
Proteins with PATRIC cross-genus family (PGfam) assignments	3,713

Abbreviations: EC, Enzyme Commission; GO, Gene Ontology database; KEGG, Kyoto Encyclopedia of Genes and Genomes.

Table S4 Antibiotic resistance related genes in the PL22-22A genome

Category	Class/subgroup	Protein	Number of related genes
β-Lactamase	Class A	CGA/CIA family	1
	Class B	MBL	13
	/	beta-lactamase family	1
Tetracycline resistance	tetracycline-inactivating enzyme	Tet(X)	1
Streptothricin resistance	Streptothricin acetyltransferase	/	1
Efflux pumps	MFS	Transporter	48
	RND	Transporter	26
	SMR	Transporter	3
	MATE	Transporter	3
	ABC	Transporter	57
Permeability defects	Porin	Outer membrane protein	14

MFS, major facilitator superfamily; RND, resistance nodulation division; SMR, small multidrug resistance; MATE, multidrug and toxic compound extrusion; ABC, ATP-binding cassette transporter.

Table S5 MIC and EC_{50} values of PMB and CST

Strains	Concentration (g/L)			
	PMB		CST	
	MIC	EC_{50}	MIC	EC_{50}
PL22-22A	96	37.35±1.102	128	61.61±1.053
DH5α (*Escherichia coli* DH5α)	<2	0.411±0.013	<2	0.534±0.02
DT *(E. coli* DH5α with pMD-18T);	<2	0.455±0.047	<2	0.509±0.02
EcAB *(E. coli* DH5α expressing *Chryseobacterium* sp. PL22-22A *cprB- cprA*)	48	19.74±1.103	32	11.99±1.063
EcABC3 *(E. coli* DH5α expressing *Chryseobacterium* sp. PL22-22A *cprABC*)	64	22.85±1.153	48	15.29±1.127

Note: The EC_{50} was presented as the mean ± SD (n = 3)

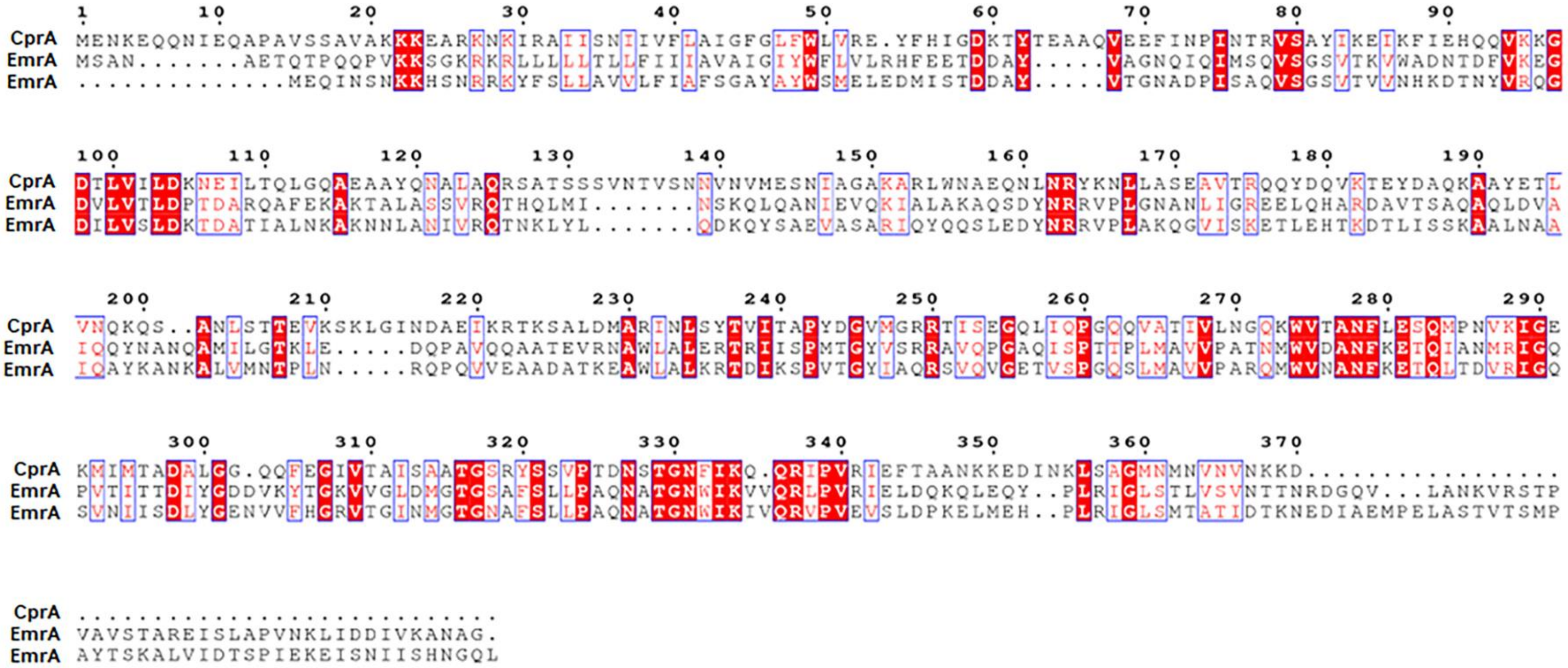

Figure S1. Alignment of amino acid sequences of CprA, EmrA and EmrK. EmrA: gene bank NO. WP_001326681.1; EmrK: gene bank NO. WP_000435167.

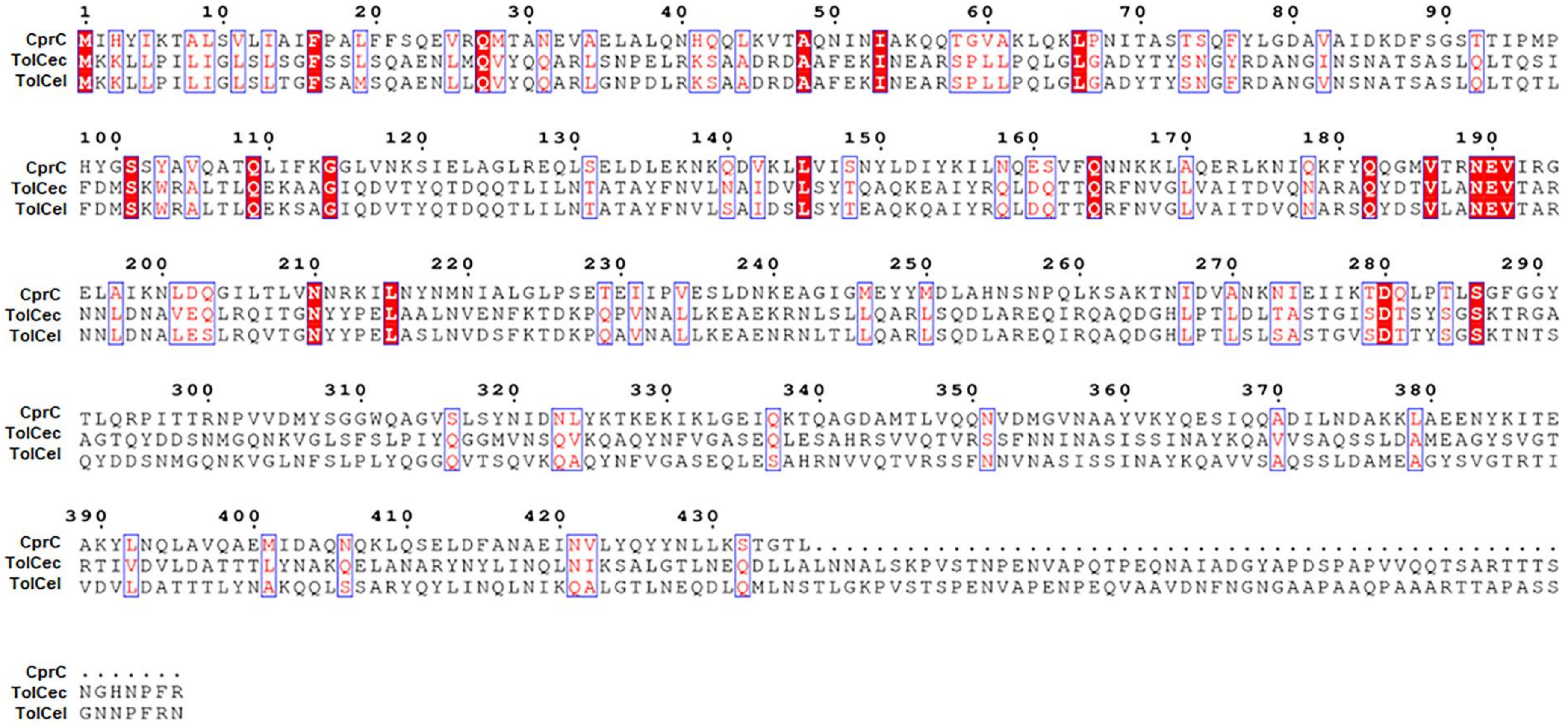

Figure S2. Alignment of amino acid sequences of CprC. CprC: TolC-like from *C. daecheongense* PL22-22A; TolCec: TolC from *E. coli* K12 (PDB: 2VDD) ; TolCel: TolC from *Enterobacter ludwigii* EcWSU1 (PDB: 5NIK)

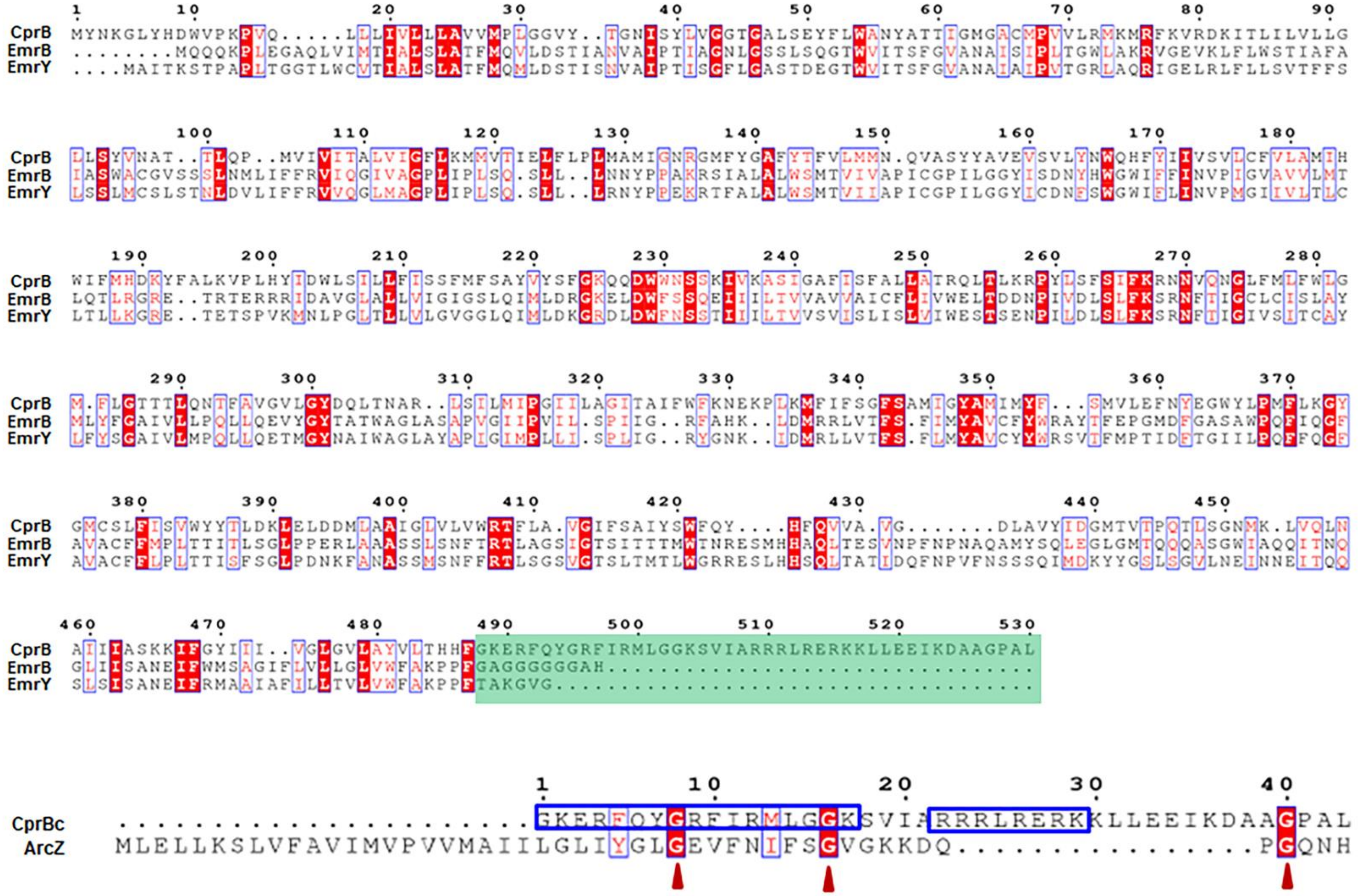

CprB
EmrB
EmrY
CprBc
ArcZ
CprBc ......................GKERFQYGRFIRMLGGKSVIARRRLRERKKLLEEIKDAAGPAL
ArcZ MLELLKSLVFAVIMVPVVMAIILGLIYGLGEVFNIFSGVGKKDQ...............PGQNH

Figure S3. Alignment of amino acid sequences of CprB, EmrB and EmrK. EmrB: gene bank NO. WP_124072429.1; EmrY: gene bank NO. EGM1824838.1. Green shadow presents the redundant sequence of CprB, compared to EmrB and EmrK.

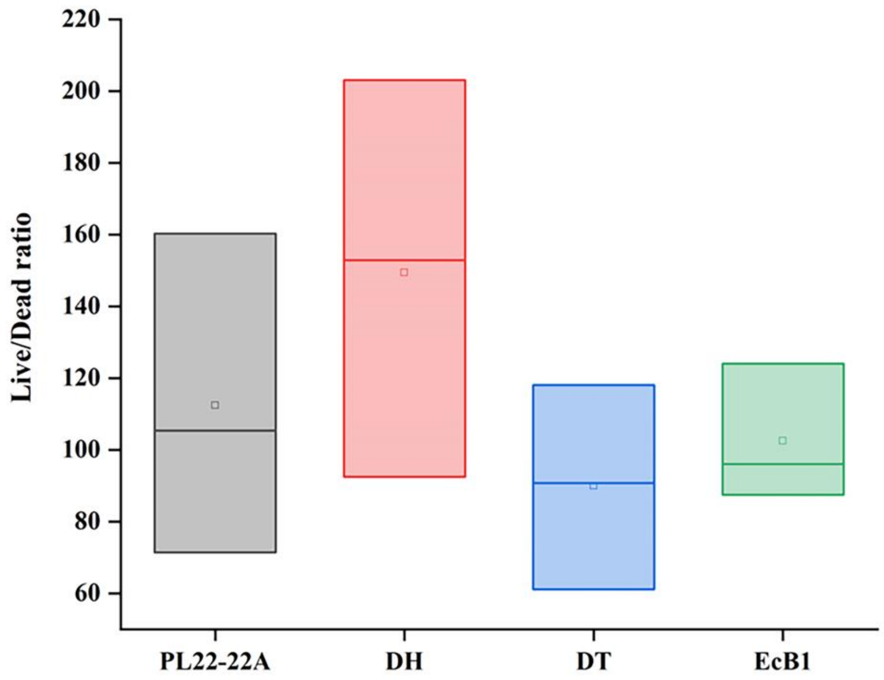

Figure S4. Viability of the bacteria in the presence of 8mg/L Baicalin. p=0.352>0.01
